# Supplementary material for: Persistent risk of hepatocellular carcinoma despite improvement of liver stiffness in patients with chronic HBV with advanced fibrosis
Source: JHEP Rep. 2025 Aug 20;7(11):101560. doi: 10.1016/j.jhepr.2025.101560 (PMC12550779; doi:10.1016/j.jhepr.2025.101560)
Supplement: Multimedia component 1 [file mmc1.pdf]

Persistent risk of hepatocellular carcinoma despite improvement of liver stiffness in patients with chronic HBV with advanced fibrosis

Lesley A. Patmore, Lilian Y Liang, George Papatheodoridis, Mai Kilany, Arno Furquim d’Almeida, Vincent WS Wong, Margarita Papatheodoridi, Thomas Vanwolleghem, Pieter Honkoop, Hans Blokzijl, Özgür M. Koc, Harry L.A. Janssen, Matthijs Kramer, Joep de Bruijne, Apichat Kaewdech, Robert A. de Man, R. Bart Takkenberg, Grace L.H. Wong, Jordan J. Feld, Milan J. Sonneveld

Table of contents

Fig. S1……………………………………………………………………………………2

Fig. S2……………………………………………………………………………………2

Fig. S3……………………………………………………………………………………3

Fig. S4……………………………………………………………………………………3

Fig. S5……………………………………………………………………………………4

Fig. S1. Flowchart of study inclusion

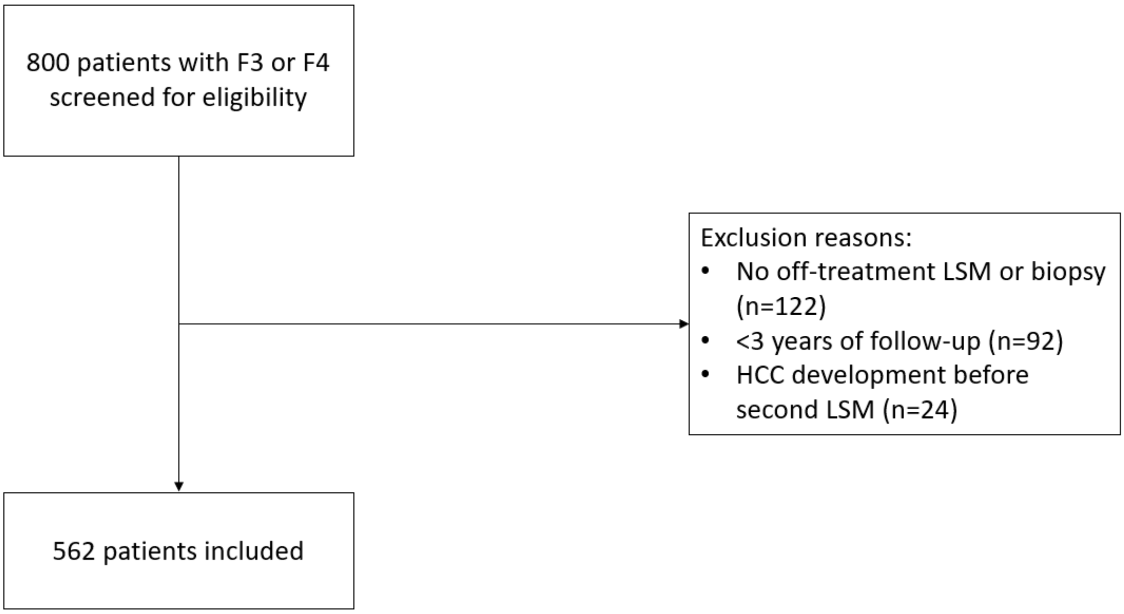

Fig. S2 Cumulative HCC incidence after on-treatment LSM in the overall cohort. *Data was analysed using the Kaplan-Meier method.*

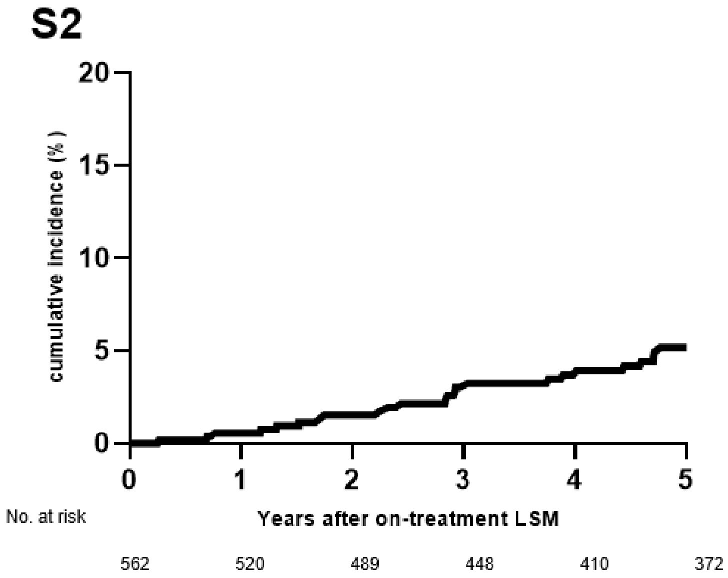

Fig. S3 Cumulative incidence of hepatic decompensation after on-treatment LSM in patients with clinically significant portal hypertension (CPSH) and no CSPH based on the BAVENO VII criteria. *Data was analysed using the Kaplan-Meier method and the log-rank test was used to compare the groups; p ≤0.05 indicaties a statistically significant difference.*

S3

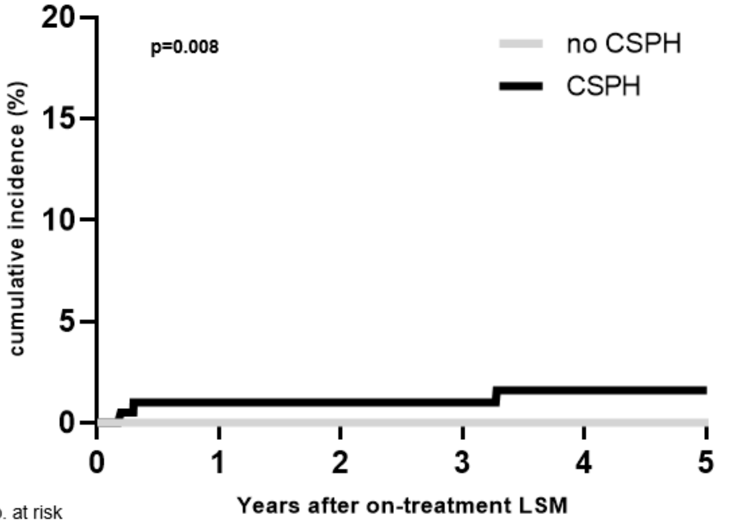

No. at risk						
CSPH	200	188	178	170	158	142
No CSPH	320	294	277	259	235	215

Fig. S4 Cumulative HCC incidence after on-treatment LSM in patients that experienced a >20% decrease in LSM compared to patients with <20% decrease in LSM. *Data was analysed using the Kaplan-Meier method and the log-rank test was used to compare the groups; p ≤0.05 indicaties a statistically significant difference.*

S4

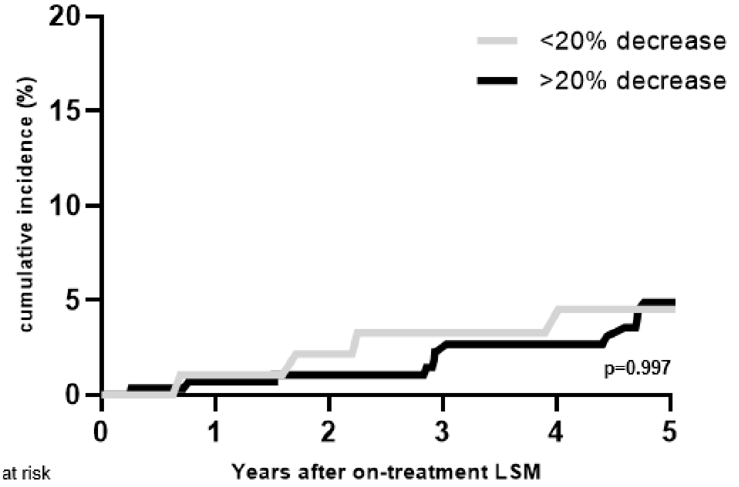

No. at risk						
<20% decrease	102	94	89	82	78	73
>20% decrease	305	277	262	242	223	212

Fig. S5A and 5B Cumulative HCC incidence after on-treatment LSM in patients eligible for HCC surveillance based on (A) PAGE-B score ≥10 and (B) pre-treatment cirrhosis. *Data was analysed using the Kaplan-Meier method and the log-rank test was used to compare the groups; p ≤0.05 indicaties a statistically significant difference.*

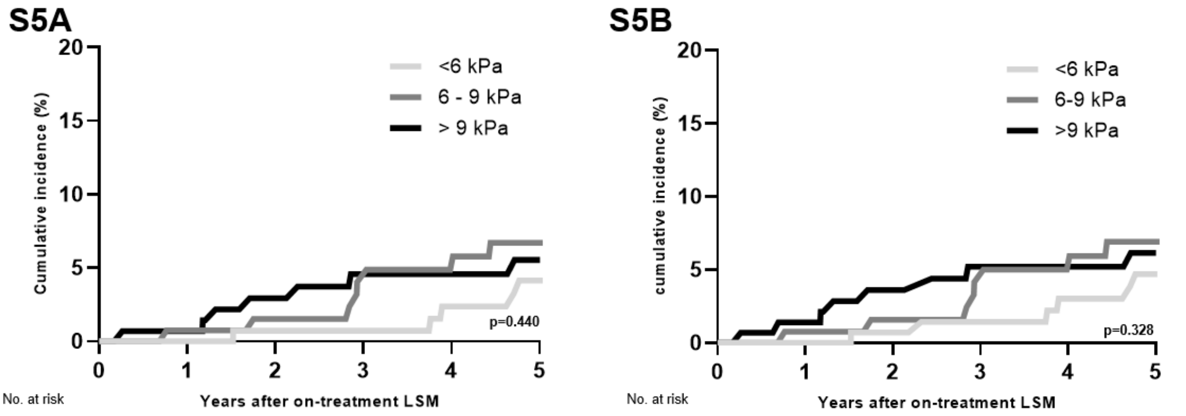

S5A

No. at risk	0	1	2	3	4	5
<6 kPa	153	147	135	130	117	107
6-9 kPa	140	130	128	115	105	97
>9 kPa	147	136	125	113	106	97

S5B

No. at risk	0	1	2	3	4	5
<6 kPa	156	148	138	131	122	112
6-9 kPa	135	125	122	112	102	94
>9 kPa	148	138	127	116	110	98
