# Supplementary material for: Persistent risk of hepatocellular carcinoma despite improvement of liver stiffness in patients with chronic HBV with advanced fibrosis
Source: JHEP Rep. 2025 Aug 20;7(11):101560. doi: 10.1016/j.jhepr.2025.101560 (PMC12550779; doi:10.1016/j.jhepr.2025.101560)
Supplement: Multimedia component 4 [file mmc4.pdf]

JHEP Reports

Persistent risk of hepatocellular carcinoma despite improvement of liver stiffness in patients with chronic HBV with advanced fibrosis

Authors

Lesley A. Patmore, Lilian Y. Liang, George Papatheodoridis, …, Grace L.H. Wong, Jordan J. Feld, Milan J. Sonneveld

Correspondence

l.patmore@erasmusmc.nl (L.A. Patmore).

Graphical abstract

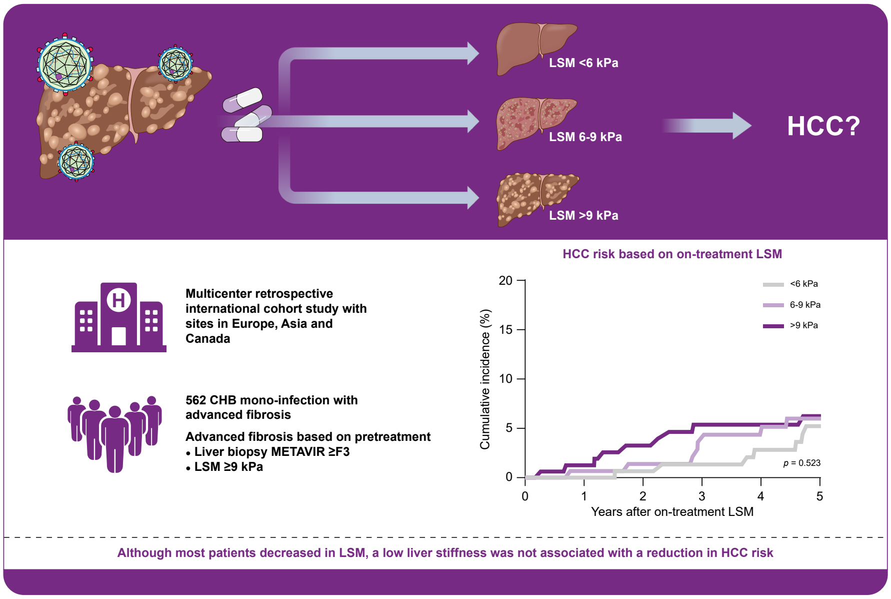

Highlights:

- Patients with CHB with advanced fibrosis are at high risk for HCC.
- Most patients with CHB experienced a decrease in LSM after initiation of antiviral therapy.
- The 5-year cumulative HCC incidence was comparable across on-treatment LSM strata.
- HCC surveillance should be continued in patients with advanced fibrosis before antiviral therapy regardless of on-treatment LSM values.

Impact and implications:

The majority of CHB patients with advanced fibrosis have a decrease in LSM during antiviral therapy. Although a decrease in LSM was associated with a lower risk of subsequent hepatic decompensation, an improvement in LSM was not associated with a reduction in HCC risk. HCC surveillance should therefore be continued in patients with advanced fibrosis at baseline regardless of LSM values obtained during therapy.

https://doi.org/10.1016/j.jhepr.2025.101560

© 2025 The Authors. Published by Elsevier B.V. on behalf of European Association for the Study of the Liver (EASL). This is an open access article under the CC BY license (http://creativecommons.org/licenses/by/4.0/).

JHEP|Reports

Persistent risk of hepatocellular carcinoma despite improvement of liver stiffness in patients with chronic HBV with advanced fibrosis

Lesley A. Patmore[1,*], Lilian Y. Liang[2], George Papatheodoridis[3], Mai Kilany[4], Arno Furquim d'Almeida[5], Vincent W.S. Wong[2], Margarita Papatheodoridi[3], Thomas Vanwolleghem[5], Pieter Honkoop[6], Hans Blokzijl[7], Özgür M. Koc[8,9], Harry L.A. Janssen[1,4], Matthijs Kramer[8,9], Joep de Bruijne[10], Apichat Kaewdech[11], Robert A. de Man[1], R. Bart Takkenberg[12], Grace L.H. Wong[2], Jordan J. Feld[4], Milan J. Sonneveld[1]

JHEP Reports **2025**. vol. 7 | 1–8

Check for updates

Background & Aims: Patients with chronic HBV (CHB) with advanced fibrosis are at high risk for hepatocellular carcinoma (HCC). Liver stiffness measurement (LSM) correlates with fibrosis in untreated patients, and is used to monitor changes in severity of liver disease. However, the association between on-treatment LSM and HCC risk is controversial.

Methods: We conducted an international multicenter retrospective cohort study of patients with CHB with advanced fibrosis and assessed the association between on-treatment LSM, HCC development, and decompensation events.

Results: We analyzed 562 patients (62.8% F4 LSM measurement, 69.2% Asian). During antiviral therapy, the on-treatment LSM decreased to <6 kPa in 209 (37.2%), to 6–9 kPa in 174 (31.0%), and remained >9 kPa in 179 (31.9%) patients. During a median follow-up of 6.8 years after the on-treatment LSM, 56 patients developed HCC and 18 (32.2%) had an on-treatment LSM <6 kPa. The 5-year cumulative HCC incidence was comparable across on-treatment LSM strata; 4.4% for <6 kPa, 5.5% for 6–9 kPa, and 5.8% for >9 kPa (p = 0.300). In multivariable analysis, older age (adjusted hazard ratio (aHR) 1.058, 95% CI 1.026–1.091, p <0.001) and lower platelet count (aHR 0.992, 95% CI 0.992–0.998, p = 0.005) were associated with HCC development, whereas on-treatment LSM was not (aHR 0.974, p = 0.974). By contrast, patients with an LSM decrease to ≤9 kPa had a negligible risk of decompensation (0% *vs.* 1.8% at 5 years, p = 0.017).

Conclusions: Most patients with CHB with advanced fibrosis experienced a decrease in LSM during antiviral therapy. Although a decrease in liver stiffness was associated with a lower risk of decompensation, an improvement in liver stiffness was not associated with a reduction in HCC risk.

© 2025 The Authors. Published by Elsevier B.V. on behalf of European Association for the Study of the Liver (EASL). This is an open access article under the CC BY license (http://creativecommons.org/licenses/by/4.0/).

Introduction

Patients with chronic HBV (CHB) with advanced fibrosis are at high risk for hepatocellular carcinoma (HCC). Studies have shown that suppression of viral replication with nucleo(s)tide analogs (NUCs) can reduce the risk of HCC, potentially through decreasing hepatic inflammation and liver fibrosis.[1,2] Therefore, monitoring changes in fibrosis grade is important in the management of patients with CHB.

Liver biopsy is considered the gold standard for the grading of fibrosis stage, but it is invasive, expensive, and associated with risks such as severe bleeding and even mortality. Therefore, liver stiffness measurement (LSM) using vibration-controlled transient elastography (VCTE) has largely replaced liver biopsy as the preferred method for assessing liver fibrosis in patients with currently untreated CHB, because LSM correlates well with liver histology in this population.[3–5] In addition, there appears to be a direct correlation between higher LSM values and HCC risk in patients with currently untreated CHB.[6,7]

Interestingly, various studies have reported an improvement in LSM after initiation of antiviral therapy in patients with CHB with advanced fibrosis, but whether this decrease in LSM reflects a reduction in severity of inflammation, fibrosis, or both, is unknown.[8–10] Furthermore, several studies have incorporated LSM obtained during treatment to predict subsequent HCC risk; however, it remains unclear to what extent a decrease in LSM in CHB with advanced fibrosis is associated with a clinically significant reduction in the risk of HCC.[11–13] Nevertheless, LSM is widely applied to monitoring changes in liver disease severity in patients with chronic liver disease, and recently updated FDA guidance for the design of trials in HBV and HDV suggest the use of non-invasive assessment of liver fibrosis, such as LSM, as surrogate endpoints in trials of novel agents.[14]

* Corresponding author. Department of Gastroenterology and Hepatology, Erasmus MC University Medical Center, Postbus 2040, 3000 CA Rotterdam, The Netherlands.
E-mail address: l.patmore@erasmusmc.nl (L.A. Patmore).
https://doi.org/10.1016/j.jhepr.2025.101560

Therefore, the aim of this study was to study changes in LSM over time in patients with CHB with advanced fibrosis initiating antiviral therapy, and to study the association between on-treatment LSM with subsequent risk of HCC and hepatic decompensation.

Patients and methods

Study design

We conducted an international multicenter retrospective cohort study of all consecutive HBV mono-infected patients with advanced fibrosis who underwent LSM at least 3 years after initiation of NUC therapy. Advanced fibrosis (METAVIR ≥F3) was diagnosed based on compatible histological findings, a pretreatment LSM ≥9.0 kPa, and/or other findings consistent with cirrhosis (*e.g.* nodular liver on imaging with signs of portal hypertension on imaging or endoscopy).

We excluded patients with a history of hepatic decompensation, presence or past viral co-infections (HDV, HCV, or HIV) or presence or development of other known chronic liver diseases (documented alcohol-related liver disease/alcohol misuse, Wilson's disease, primary biliary cholangitis, primary sclerosing cholangitis, autoimmune hepatitis, and hemochromatosis). Patients who did not start antiviral therapy with a NUC or who developed HCC or hepatic decompensation before the on-treatment LSM were also excluded.

Data collection and endpoint definitions

Data were collected from academic and non-academic sites in Belgium, Canada, Greece, Hong Kong, the Netherlands, and Thailand. Baseline was set as the date of the first off-therapy fibrosis assessment that met the inclusion criteria. An LSM >12 kPa was considered as cirrhosis. Data at on-treatment LSM were obtained at the time of the first on-treatment LSM performed after at least 3 years of follow-up. LSM was obtained by VCTE (Fibroscan, Echosens, Paris, France; equipped with both M and XL probes) by trained healthcare providers. Only examinations with at least 10 valid individual measurements and acceptable variances, defined as IQR <30%, were deemed valid. On-treatment LSM values were categorized according to cut-offs validated against METAVIR stage in untreated patients; <6 kPa (F0-1), 6–9 kPa (F2) and >9 kPa (F3–4).[15]

Diagnosis of HCC was based on compatible imaging findings or histology, as per clinical guidelines. Decompensation was defined as the first of a composite of variceal bleeding, clinically relevant ascites that required the use of diuretics, or large volume paracentesis or overt hepatic encephalopathy that required treatment.

Statistical analysis

The primary aim of the study was assessment of the cumulative incidence of HCC across on-treatment LSM strata and according to LSM-based risk scores. The key secondary aim was to study the cumulative incidence of hepatic decompensation across on-treatment LSM strata and according to BAVENO VII criteria for ruling out clinically significant portal hypertension (*i.e.* LSM <15 kPa with platelet count $>150\times10^6$/L).[16] Continuous variables were described using median and IQR or mean values and SD. Categorical variables were described as frequencies or percentages. The CAGE-B (based on baseline presence of cirrhosis, age, and follow-up LSM) and the SAGE-B (based on age and LSM at follow-up) scores were calculated as previously reported,[11] and patients were classified as low (<6), intermediate (6–10) or high (>10) risk for HCC development based on previously reported cut-offs.[11] The cumulative incidence of HCC and decompensation after the on-treatment LSM was assessed using the Kaplan-Meier method and compared between different groups using the log-rank test. Follow-up time was calculated as the interval between the date of the on-treatment (follow-up) LSM and the date of the event of interest (HCC or hepatic decompensation). Univariable Cox regression models were used to estimate the effect of various variables on the hazard of HCC and hepatic decompensation. Multivariable Cox regression was used to identify independent prognostic factors. Hazard ratios (HRs) and their 95% CIs along with corresponding p values are presented. A p value <0.05 was considered to be statistically significant. All data were analyzed using IBM SPSS Statistics Version 28.

Ethics

This study was conducted in accordance with the guidelines of the Declaration of Helsinki and the principles of Good Clinical Practice. The study protocols were primarily reviewed by the Erasmus MC Medical Ethical Committee. The requirement for informed consent was waived, and the institutional review boards of participating sites gave necessary approval whenever required.

Results

Patient characteristics at baseline (pretreatment) fibrosis assessment

We screened 800 patients for eligibility; 122 patients were excluded because they had no off-treatment LSM or biopsy; 92 patients had <3 years of follow-up; and 24 developed HCC before the on-treatment LSM (Fig. S1). We included and analyzed 562 patients with a median age at baseline of 50 years (IQR 41–58), 75.6% were men. Most of the patients were Asian (69.2%) or Caucasian (21.7%). Median alanine aminotransferase (ALT) was 57 U/L (IQR 38–109), median platelet count was 179 × 10^9/L (IQR 142–229) and median HBV DNA was 5.5 $\log_{10}$ IU/ml (IQR 3.9–6.6). At baseline, 209 (37.2%) of the patients had F3 and 353 (62.8%) had F4 based on histology (15.7%) or LSM (65.3%). Among patients with baseline LSM data (n = 367, 65.3%), the median baseline LSM was 12.5 kPa, (IQR 10.5–17.1). Information on the presence of metabolic comorbidities was available for a subset of patients; 95 patients (16.9%) had diabetes, 160 (28.5%) had hypertension, 58 (10.3%) had dyslipidemia, and 100 (17.8%) had steatosis. All patients initiated antiviral therapy with a NUC; most were treated with entecavir (ETV; 52%). All patient characteristics at baseline are described in Table 1.

Patient characteristics at on-treatment LSM

All patients underwent an on-treatment LSM. The median time between baseline fibrosis assessment and on-treatment LSM was 4.7 years (IQR 3.6–6.6) and median on-treatment LSM was 7.1 kPa (IQR 5.1–10.3). On-treatment LSM was <6 kPa in 209 (37.2%) patients, 6–9 kPa in 174 (31.0%) patients, and

Table 1. Baseline characteristics.

At start of therapy (n = 562)	
Age, years	50 (41–58)
Male, n (%)	425 (75.6)
Ethnicity, n (%)	
Asian	389 (69.2)
Caucasian	122 (21.7)
Other	51 (9.7)
ALT, U/L	57 (38–109)
Platelet count, × 10^9/L	179 (142–229)
HBeAg, n (%)	191 (34)
HBV DNA, $\log_{10}$ IU/ml	5.5 (3.9–6.6)
Fibrosis n (%)/(biopsy, n/LSM, n)	
F3	209 (37.2)/(64/145)
F4	353 (62.8)/(81/222)
Initial antiviral therapy, n (%)	
ETV	292 (52.0)
TDF/TAF	123 (21.9)
Other	147 (26.2)
Diabetes, n (%)	95 (16.9)
Hypertension, n (%)	160 (28.5)
Dyslipidemia, n (%)	58 (10.3)
Overweight, n (%)	111 (19.8)
Steatosis, n (%)	100 (17.8)
At on-treatment LSM (n = 562)	
Median time between baseline and on-treatment LSM, years	4.7 (3.6–6.6)
Age, years	56 (47–63)
ALT, U/L	28 (21–41)
Platelet count, ×10^9/L	173 (139–214)
Undetectable HBV DNA, n (%)	472 (84)
LSM, n (%)	
<6 kPa	209 (37.2)
6–9 kPa	174 (31.0)
>9 kPa	179 (31.9)
Antiviral therapy, n (%)	
ETV	298 (53.0)
TDF/TAF	156 (27.8)
Other	108 (19.1)
**End of follow-up**	
Median follow-up time after on-treatment LSM, years	6.4 (3.7–11.1)
HCC, n (%)	56 (10.4)
Decompensation, n (%)	16 (2.8)

Data are presented as mean (SD) for normally distributed data, as median (IQR) for non-normally distributed data and as n(%) for categorical variables. *Some patients received multiple types of antiviral therapy. ALT, alanine aminotransferase; ETV, entecavir; HCC, hepatocellular carcinoma; LSM, liver stiffness measurement; TAF, tenofovir alafenamide fumarate; TDF, tenofovir disoproxil fumarate.

>9 kPa in 179 (31.9%) patients. The median ALT at on-treatment LSM was 28 U/L (IQR 21–41) and 84% had undetectable HBV DNA at on-treatment LSM. All patient characteristics at on-treatment LSM are described in Table 1.

HCC development in the overall cohort and stratified by LSM

During a median follow-up of 6.4 years (IQR 3.7–11.1) after the on-treatment LSM, 56 patients developed HCC. The 5- and 10-year cumulative incidences of HCC were 5.2% and 14.1% (Fig. S2). There was no significant difference in HCC risk between Asian and non-Asian patients (4.9% *vs.* 5.4% at 5 years, p = 0.675).

In univariable analysis, older age (HR 1.065, 95% CI 1.038–1.093, p <0.001), lower platelet count (HR 0.990, 95% CI 0.985–0.996, p <0.001), diabetes mellitus (HR 2.393, 95% CI 1.347–4.251, p = 0.003), and hypertension (HR 3.352, 95% CI 1.910–5.884, p <0.001) were predictors for HCC development, whereas on-treatment LSM was not (HR 1.016, 95% CI 0.983–1.050, p = 0.357; Table 2).

Of the 56 patients who developed HCC after the on-treatment LSM, 18 (32.2%) had an on-treatment LSM <6 kPa, 19 (33.9%) had an on-treatment LSM 6–9 kPa, and 19 (33.9%) had an on-treatment LSM >9 kPa. The 5-year cumulative HCC incidence was comparable across on-treatment LSM strata; 4.4% for <6 kPa, 5.5% for 6–9 kPa, and 5.8% for >9 kPa (p = 0.300, Fig. 1). Findings were consistent among the subset of patients enrolled in this study based on either advanced fibrosis diagnosed on liver biopsy (n = 145, p = 0.371) or non-invasive assessment (n = 417, p = 0.146).

In a multivariable analysis adjusting for sex, baseline fibrosis status, diabetes mellitus, and hypertension, only older age (adjusted HR [aHR] 1.058, 95% CI 1.026–1.091, p <0.001) and lower platelet count (aHR 0.992, 95% CI 0.992–0.998, p = 0.005) were significantly associated with HCC development, whereas on-treatment LSM was not (aHR 0.974, 95% CI 0.932–1.018, p = 0.974) (Table 2).

Performance of the CAGE-B and SAGE-B score for the prediction of HCC

The median CAGE-B score was 9 (IQR 6–11), and 102 (18.1%), 288 (51.2%), and 172 (30.6%) patients were classified as low, intermediate, and high risk, respectively, based on their CAGE-B score. The 5-year cumulative incidence of HCC in the low-risk CAGE-B group was 0%, compared with 5.9% in the intermediate-risk group and 6.7% in the high-risk group (p = 0.002 by log-rank test; Fig. 2A).

The median SAGE-B risk was 6 (IQR 4–9), and 158 (28.1%), 325 (57.8%), and 79 (14.1%) patients were classified as low, intermediate, and high risk, respectively, based on their SAGE-B score. The 5-year cumulative incidence of HCC in the low-risk SAGE-B group was 2.3%, compared with 5.5% in the

Table 2. Univariable and multivariable analysis of factors associated with incident HCC.

	Univariable			Multivariable		
	HR	95% CI	*p* value	aHR	95% CI	*p* value
Age, years	1.065	1.038–1.093	<0.001	1.058	1.026–1.091	<0.001
Male sex	0.984	0.529–1.831	0.960	1.155	0.598–2.230	0.667
Platelet count, ×10^9/L	0.990	0.985–0.996	<0.001	0.992	0.992–0.998	0.005
Baseline F4	1.368	0.692–2.703	0.367	1.244	0.676–2.289	0.483
Diabetes	2.393	1.347–4.251	0.003	1.209	0.646–2.260	0.553
Hypertension	3.352	1.910–5.884	<0.001	1.816	0.971–3.395	0.062
On-treatment LSM, kPa	1.016	0.983–1.050	0.357	0.974	0.932–1.018	0.974

Results were obtained with Cox proportional hazard analysis and given as (a)HR with 95% CI and p value. aHR, adjusted hazard ratio; HCC, hepatocellular carcinoma; HR, hazard ratio; LSM, liver stiffness measurement.

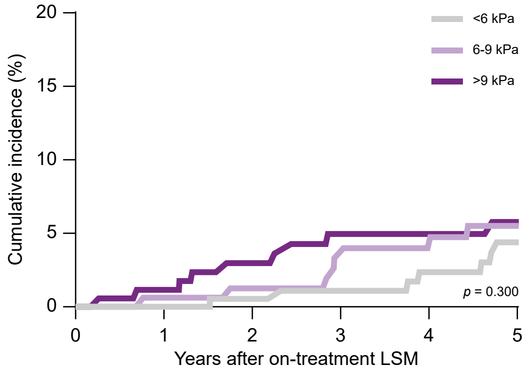

No. at risk						
<6 kPa	209	194	181	170	153	139
6-9 kPa	174	159	155	141	128	117
>9 kPa	179	167	153	137	129	116

Fig. 1. Cumulative HCC incidence after on-treatment LSM stratified by on-treatment LSM category. Data were analyzed using the Kaplan-Meier method and the log-rank test was used to compare the groups; *p* ≤0.05 indicates a statistically significant difference. HCC, hepatocellular carcinoma; LSM, liver stiffness measurement.

intermediate-risk group and 8.9% in the high-risk group (*p* = 0.013 by log-rank test; Fig. 2B).

Importantly, the association between CAGE-B/SAGE-B scores and HCC appeared to be predominantly driven by age. We observed no HCC in the subset of patients aged <40 years at follow-up LSM, compared with a 5-year cumulative HCC incidence of 5.8% for patients aged >40 years (*p* = 0.024; Fig. 3A). Therefore, we performed a sensitivity analysis in a subset of patients older than 40 years at on-treatment LSM. Among these 490 patients, 170 (34.7%) patients had an on-treatment LSM <6 kPa, 157 (33.4%) patients had 6–9 kPa, and 163 (33.3%) patients had >9 kPa. The 5-year cumulative HCC incidence was comparable across on-treatment LSM strata; 5.2% for <6 kPa, 6.0% for 6–9 kPa, and 6.2% for >9 kPa (*p* = 0.523, Fig. 3B).

Decompensation events in the overall cohort and stratified by LSM

After the on-treatment LSM, 16 patients experienced hepatic decompensation (seven developed hepatic decompensation, six developed variceal bleed, and three developed hepatic encephalopathy). In 10 of the 16 patients, the decompensation event was preceded by the development of HCC.

The 5-year cumulative incidence of decompensation was 0.6%. In univariable analysis, older age (HR 1.074, 95% CI 1.021–1.130, *p* = 0.006) and on-treatment LSM (HR 1.047, 95% CI 1.004–1.091, *p* = 0.034) were predictors for decompensation. Given the low number of events, no multivariable analysis was performed.

We found no decompensation events in patients with an on-treatment LSM <9 kPa and, therefore, we combined patients from the <6 kPa group with the 6–9 kPa group. The cumulative incidence of hepatic decompensation for patients with <9 kPa at the on-treatment LSM (n = 381) was 0%, compared with the 1.8% for patients with an on-treatment LSM >9 kPa (*p* = 0.017; Fig. 4). Findings were consistent after excluding patients with a decompensation event after HCC development (*p* = 0.002). In 317 (56.4%) patients, clinically significant portal hypertension could be ruled out based on BAVENO VII criteria. No cases of hepatic decompensation were observed in this group (*p* = 0.008; Fig. S3).

Discussion

In this study of 562 patients with previously untreated CHB with advanced fibrosis enrolled from 11 centers in Asia, Europe, and North America, we observed that most patients experienced a marked improvement in LSM after initiation of antiviral therapy. Although a reduction in LMS to ≤9 kPa was associated with a reduced risk of hepatic decompensation, we observed a persistent risk of HCC in this population. These findings suggest that on-treatment LSM values should not be used to guide HCC surveillance strategies.

The current study provides important insights into the risk of liver-related events in patients with CHB with advanced

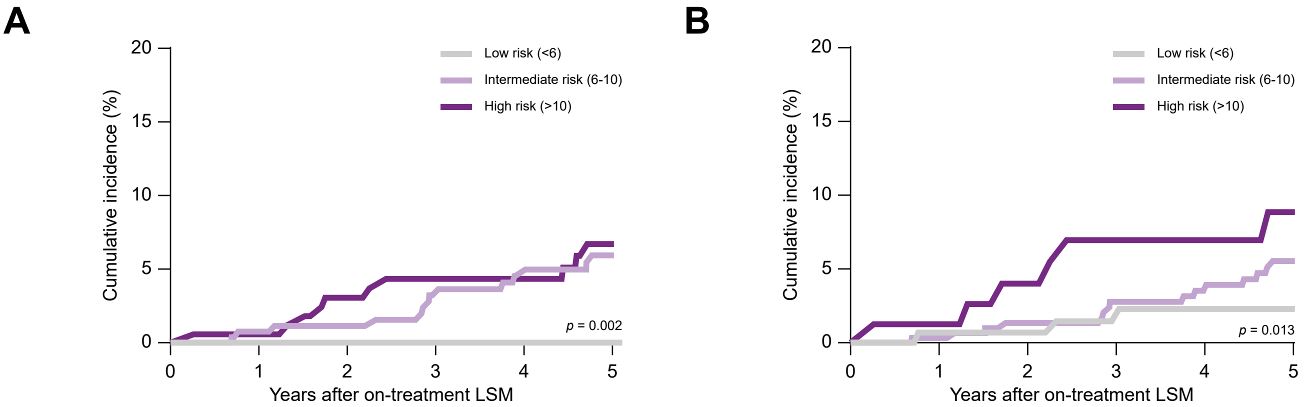

A

No. at risk						
Low	102	95	90	77	66	61
Intermediate	288	260	244	231	212	196
High	172	165	155	140	132	115

B

No. at risk						
Low	158	142	134	119	103	94
Intermediate	325	302	287	271	251	230
High	79	76	68	58	56	48

Fig. 2. Cumulative HCC incidence after on-treatment LSM stratified by (A) CAGE-B strata and (B) SAGE-B strata. Data were analyzed using the Kaplan-Meier method and the log-rank test was used to compare the groups; *p* ≤0.05 indicates a statistically significant difference. HCC, hepatocellular carcinoma; LSM, liver stiffness measurement.

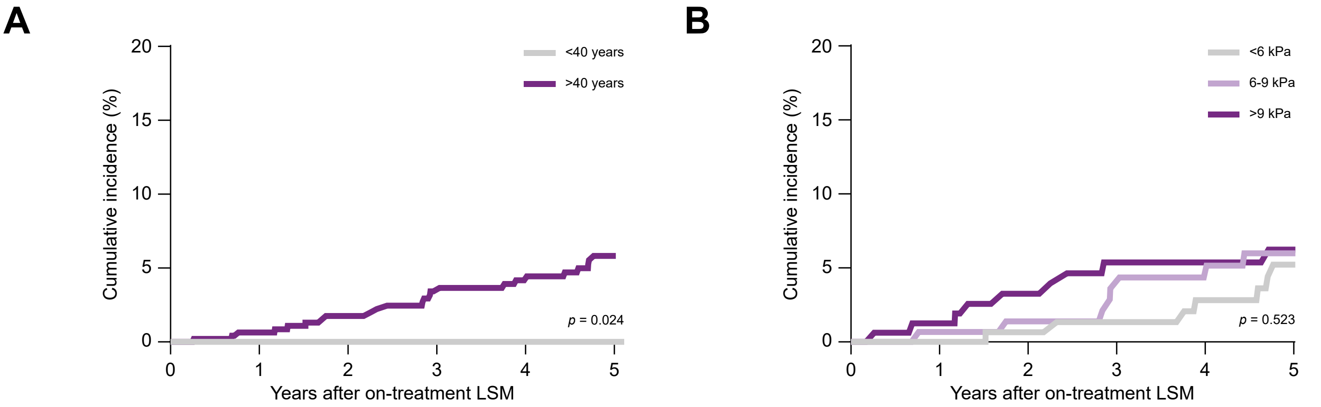

No. at risk						
<40 years	72	64	49	40	38	32
>40 years	490	456	430	400	370	334

No. at risk						
<6 kPa	170	160	148	141	130	117
6-9 kPa	157	144	140	130	118	108
>9 kPa	163	152	142	128	122	109

Fig. 3. Cumulative HCC incidence after on-treatment LSM stratified by (A) age and (B) on-treatment LSM strata in the subset of patients >40 years. Data were analyzed using the Kaplan-Meier method and the log-rank test was used to compare the groups; *p* ≤0.05 indicates a statistically significant difference. HCC, hepatocellular carcinoma; LSM, liver stiffness measurement.

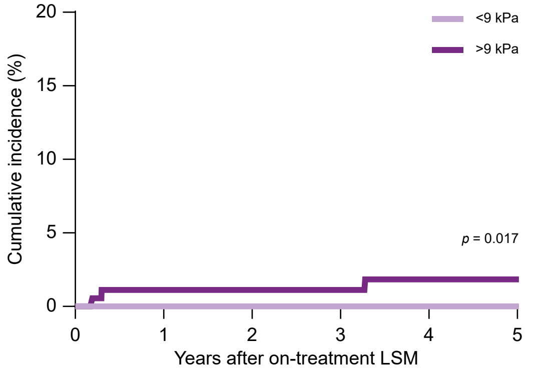

No. at risk						
≤9 kPa	381	352	337	316	286	263
>9 kPa	182	168	155	146	136	122

Fig. 4. Cumulative incidence of hepatic decompensation after on-treatment LSM stratified by LSM ≤9 kPa and LSM >9 kPa. Data were analyzed using the Kaplan-Meier method and the log-rank test was used to compare the groups; *p* ≤0.05 indicates a statistically significant difference. LSM, liver stiffness measurement.

fibrosis who are treated with NUCs. First of all, the risk of *de novo* decompensation events appears to be very low; the 5-year cumulative incidence after the follow-up LSM assessment was 0.6%. Unfortunately, our study also highlights the persistent risk of HCC development in this population; the 5- and 10-year cumulative HCC incidences were 5.2% and 14.1%. These observations are in line with previous reports and underscore that HBV DNA suppression alone is insufficient to eliminate HCC risk.[17] Based on these findings, exploration of alternative strategies to increase rates of functional cure is paramount if the risk of HCC is to be mitigated. Pending availability of such options, our findings support current recommendations to continue HCC surveillance in this patient group despite viral suppression.

The high persistent risk of HCC remains disappointing in the light of previous data suggesting that reversal of cirrhosis is possible in most patients with cirrhosis treated with ETV or tenofovir disoproxil fumarate (TDF).[1] Based on these observations, we hypothesized that a reduction in fibrosis stage, assessed using LSM, would also reflect a reduction in HCC risk. In the current cohort, 77.4% had an on-treatment LSM suggestive of fibrosis improvement, a number that is remarkably similar to the proportion reported by Marcellin *et al.* using histology as a reference.[1] Nevertheless, LSM can be influenced by a myriad of factors beyond liver fibrosis, such as inflammation, cholestasis, and cardiac congestion.[18]

There are multiple potential explanations for our findings. First, it is unclear whether an on-treatment reduction in LSM reflects a decrease in inflammatory activity, a decrease in liver fibrosis severity, or both. As such, a significant proportion of patients with an improvement in LSM might not have achieved fibrosis regression, which could account for the failure to achieve a significant reduction in HCC risk. Another potential explanation pertains to the fact that many oncogenic processes associated with HBV infection, including HBV DNA integration, have already occurred before therapy initiation and might not be reversible even with long-term treatment. In this scenario, reductions in fibrosis stage, even if confirmed with liver biopsy, might not be sufficient to completely eliminate the risk of HCC.

Our results do not appear to confirm the findings from a recent follow-up analysis of the PAGE-B cohort, in which Papatheodoridis *et al.* compared the cumulative incidence of HCC beyond 5 years of therapy with ETV or TDF in White patients with CHB according to fibrosis status at baseline and the LSM at year 5.[11] In this study, higher follow-up LSM values were linked to a statistically higher HCC risk during subsequent follow-up. However, careful assessment showed that, although HCC might have occurred somewhat earlier in patients with higher LSM, HCC was observed in 4.9% of patients

with cirrhosis at baseline who had a decrease in LSM to <12 kPa.[11] Therefore, these findings are consistent with the current report. Recently, Semmler *et al.* investigated dynamic changes in LSM in patients with advanced chronic liver disease. They reported that a reduction of at least 20% in LSM was associated with a lower risk of hepatic decompensation. However, their study population included patients with various underlying liver disease etiologies, only a small proportion of whom had CHB, as well as shorter intervals between LSM assessments and different clinical endpoints. These differences limit direct comparability with our cohort. In our study, 305 patients experienced a ≥20% decrease in LSM, but showed a similar 5-year incidence of HCC compared with those without such a decrease (4.5% *vs.* 4.9%, p = 0.997; Fig. S4).

We also attempted to validate two proposed risk scores, CAGE-B and SAGE-B, for prediction of HCC risk after the follow-up LSM. Both risk scores include age and LSM after 5 years of treatment. In our cohort, HCC risk was higher in patients with higher CAGE-B and SAGE-B scores, but this appeared to be driven by age, not by LSM. In our cohort, all HCCs developed in patients over 40 years of age at the time of follow-up LSM. Therefore, we performed a sensitivity analysis in patients >40 years at on-treatment LSM and found comparable cumulative incidences across different on-treatment LSM strata. These discrepant findings might be explained by differences in patient and viral characteristics, given that the PAGE-B cohort comprised a White population and the current cohort was predominantly Asian patients.

Our findings could have important clinical implications. Given the persistent HCC risk, continued HCC surveillance is recommended despite successful HBV DNA suppression among patients with CHB with advanced fibrosis. As the HBV population ages, the number of patients eligible for HCC surveillance will increase rapidly.[19] Although baseline risk scores, such as PAGE-B, might be valuable in stratifying HCC risk, the validity in patients with advanced fibrosis is uncertain, and clinical application is hampered by the fact that only a few patients with cirrhosis have low PAGE-B scores.[20–22] As such, alternative methods are urgently needed. The current study suggests that a reduction in LSM to values associated with the absence of fibrosis in untreated patients is not associated with elimination of HCC risk and, therefore, that surveillance should be continued in patients with advanced fibrosis at baseline regardless of LSM values obtained during therapy. Furthermore, our findings also indicate that on-treatment LSM could have value in predicting subsequent decompensation events in this population.

Although this is a unique cohort comprising a mixed-ethnicity population obtained from expert centers across the globe, our study has limitations. First, this is an international retrospective cohort study in which not all potential risk factors for the development of HCC, use of moderate alcohol consumption (patients with alcohol misuse were excluded), smoking, and other lifestyle factors could be taken into account. In addition, the decisions to start treatment and when to perform LSM varied by site and country. However, sensitivity analyses revealed no influence of center on the study findings. Second, some patients used older antiviral agents, such as lamivudine or adefovir, potentially increasing the risk of HCC development. However, most started treatment with ETV or tenofovir, and most of those who did not initially use these antivirals transitioned to them before the on-treatment LSM or during follow-up. We found similar results in a sensitivity analysis excluding patients on antiviral therapy other than ETV or tenofovir. Furthermore, detectable HBV DNA at on-treatment LSM was not associated with an increased risk of HCC and excluding patients with detectable HBV DNA at follow-up LSM also did not influence any of the findings. Third, liver stiffness thresholds remain controversial and variable across studies. LSM values of >8–9 kPa are typically associated with the presence of significant or advanced fibrosis.[4,23] For the current study, we used a cut-off of 9 kPa to enrich our study population for patients with more severe liver disease. Unfortunately, we were unable to determine whether LSM in our cohort correlated accurately with histological stages F3 or F4, because no paired liver biopsies were performed for validation. Furthermore, with the current dataset, we were unable to additionally calculate other fibrosis scores, such as the enhanced liver fibrosis score. However, existing literature has consistently demonstrated that, in treatment-naïve patients, LSM values corresponding to F3 and F4 stages generally show good concordance with histological fibrosis grading.[3] Moreover, there is currently no established evidence to determine the optimal timing for on-treatment fibrosis assessment after initiation of antiviral therapy. Therefore, we selected a pragmatic cut-off of a minimum of 3 years between the first and second assessments. To further ensure the robustness of our findings, we conducted a subanalysis including only patients with at least a 5-year interval between fibrosis measurements and found consistent results: the 5-year HCC risk was 4.0% for patients with LSM <6 kPa, 3.8% for those with LSM 6–9 kPa, and 5.2% for those with LSM >9 kPa (p = 0.800). However, long-term follow-up after the on-treatment LSM, preferably with multiple LSMs, is needed to confirm our findings. In addition, most of the sites were academic centers, which could influence the external validity of our findings. In our cohort, 90.7% of patients were eligible for HCC surveillance based on the presence of cirrhosis, PAGE-B ≥10, or other criteria. Findings were consistent when limiting our analyses to patients eligible for HCC surveillance based on these criteria (Fig. S5A and B). Finally, although we observed a lower risk of decompensation events in patients with a follow-up LSM ≤9 kPa, the number of events was small and, therefore, this finding requires further confirmation.

In conclusion, most patients with CHB with advanced fibrosis experienced a decrease in LSM during antiviral therapy. Unfortunately, although this improvement in LSM was associated with a reduced risk of subsequent decompensation events, it was not associated with a clinically relevant reduction in the risk of HCC. Therefore, baseline fibrosis stage and not on-treatment LSM should be used to guide HCC surveillance strategies.

Affiliations

[1]Department of Gastroenterology and Hepatology, Erasmus MC, University Medical Center, Rotterdam, The Netherlands; [2]Medical Data Analytics Centre, Department of Medicine and Therapeutics, The Chinese University of Hong Kong, Hong Kong Special Administrative Region of China; [3]Department of Gastroenterology, School of Medicine, National and Kapodistrian University of Athens, Laiko General Hospital Athens, Athens, Greece; [4]Toronto Centre for Liver Disease, Toronto General Hospital, University of Toronto, Toronto, ONT, Canada; [5]Department of Gastroenterology and Hepatology, Antwerp University Hospital, Antwerp, Belgium; [6]Department of Gastroenterology and Hepatology, Albert Schweitzer Hospital, Dordrecht, The Netherlands; [7]Department of Gastroenterology and Hepatology, University Medical Center Groningen, University of Groningen, Groningen, The Netherlands; [8]Department of Gastroenterology and Hepatology, Maastricht University Medical Center, Maastricht, The Netherlands; [9]GROW–School for Oncology & Reproduction, Maastricht University, Maastricht, The Netherlands; [10]Department of Gastroenterology and Hepatology, Utrecht UMC, Utrecht, The Netherlands; [11]Gastroenterology and Hepatology Unit, Division of Internal Medicine, Faculty of Medicine, Prince of Songkla University, Songkhla, Thailand; [12]Department of Gastroenterology and Hepatology, Amsterdam University Medical Centers, Amsterdam, The Netherlands

Abbreviations

aHR, adjusted hazard ratio; ALT, alanine aminotransferase; CI, confidence interval; CHB, chronic HBV; ETV, entecavir; HCC, hepatocellular carcinoma; HR, hazard ratio; IQR, interquartile range; LSM, liver stiffness measurement; NUC, nucleo(s)tide analog; TAF, tenofovir alafenamide fumarate; TDF, tenofovir disoproxil fumarate; VCTE, vibration-controlled transient elastography.

Financial support

The study was sponsored by the Foundation for Liver and Gastrointestinal Research (SLO), Rotterdam, The Netherlands. The SLO had no influence on study design, data acquisition or analysis, or the decision to submit for publication.

Conflicts of interest

LAP: research grant from Gilead Sciences. GP: advisor/lecturer for AbbVie, Albireo, Amgen, Dicerna, and Genesis; research grant from Gilead Sciences; member of data safety monitoring board or advisory board for Gilead Sciences, Novo Nordisk, Janssen, Ipsen, GSK, and Roche. VW: consultant or advisory board member for AbbVie, Boehringer Ingelheim, Echosens, Gilead Sciences, Intercept, Inventiva, Novo Nordisk, Pfizer, Sagimet Biosciences, TARGET PharmaSolutions, and Visirna; speaker for Abbott, AbbVie, Echosens, Gilead Sciences, Novo Nordisk, and Unilab; research grant from Gilead Sciences; co-founded Illuminatio Medical Technology. TV: senior clinical investigator fellowship from the Research Foundation Flanders (FWO) (18B2821N). His institution has received grants from GSK, Gilead Sciences, Bristol Myers Squibb, and Fujirebio. TVW: consultant for MSD, Janssen Pharmaceuticals, Gilead Sciences, AbbVie, Bristol Myers Squibb; sponsored lectures for Gilead Sciences, and AbbVie; received support for attending conferences/travel from Ipsen, Gilead and AbbVie. HLAJ: research grants from Gilead Sciences, GSK, Janssen, Roche, Vir Biotechnology; consultant for Aligos, Gilead Sciences, GSK, Grifols, Roche, Vir Biotechnology Inc, and Precision Biosciences. JdB: research support from Terumo. AK: research grants or support from Roche, Roche Diagnostics, and Abbott Laboratories, and honoraria from Roche, Roche Diagnostics, Abbott Laboratories, and Esai. RAdM: research support from Roche. RBT: research support from Norgine, speaker fees from WL Gore and Falk; consultant for Gilead, Boehringer Ingelheim, Astra Zenica, and 89Bio. GLHW: research grants from Gilead Sciences; consultant for AstraZeneca, Gilead Sciences, GSK, and Janssen; speaker fees from Abbott, AbbVie, Ascletis, Bristol Myers Squibb, Echosens, Gilead Sciences, Janssen, and Roche; JJF: personal fees for consulting from AbbVie, Enanta, Gilead, Janssen, and Roche, and research funding from AbbVie, Abbott, Gilead, and FUJIFILM Wako Chemicals. MJS: research support and consultancy from Roche, Gilead, Albireo/Ipsen, Fujirebio. None of the remaining authors have any interests to declare.

Please refer to the accompanying ICMJE disclosure forms for further details.

Authors' contributions

Collection of data: LAP, LYL, MK, AK. Study design, writing of manuscript and approval of final version: LAP, MJS. Critical review of the manuscript and approval of final version: LYL, GP, MKilany, AFA, VW, MP, TV, PH, HB, OMK, HLAJ, MKramer, JdB, AK, RAdM, RBT, GLW, JJF.

Data availability

The data that support these findings are not publicly available, because they are subject to (inter)national data protection laws to ensure data privacy of the study participants. Therefore, the data cannot be shared.

Supplementary data

Supplementary data to this article can be found online at https://doi.org/10.1016/j.jhepr.2025.101560.

References

[1] Marcellin P, Gane E, Buti M, et al. Regression of cirrhosis during treatment with tenofovir disoproxil fumarate for chronic hepatitis B: a 5-year open-label follow-up study. Lancet 2013;381:468–475.

[2] Papatheodoridis GV, Lampertico P, Manolakopoulos S, et al. Incidence of hepatocellular carcinoma in chronic hepatitis B patients receiving nucleos(t)ide therapy: a systematic review. J Hepatol 2010;53:348–356.

[3] Marcellin P, Ziol M, Bedossa P, et al. Non-invasive assessment of liver fibrosis by stiffness measurement in patients with chronic hepatitis B. Liver Int 2009;29:242–247.

[4] Li Y, Huang YS, Wang ZZ, et al. Systematic review with meta-analysis: the diagnostic accuracy of transient elastography for the staging of liver fibrosis in patients with chronic hepatitis B. Aliment Pharmacol Ther 2016;43:458–469.

[5] Seo YS, Kim MY, Kim SU, et al. Accuracy of transient elastography in assessing liver fibrosis in chronic viral hepatitis: a multicentre, retrospective study. Liver Int 2015;35:2246–2255.

[6] Fung J, Lai CL, Seto WK, et al. Prognostic significance of liver stiffness for hepatocellular carcinoma and mortality in HBeAg-negative chronic hepatitis B. J Viral Hepat 2011;18:738–744.

[7] Chon YE, Jung ES, Park JY, et al. The accuracy of noninvasive methods in predicting the development of hepatocellular carcinoma and hepatic decompensation in patients with chronic hepatitis B. J Clin Gastroenterol 2012;46:518–525.

[8] Kim MN, Kim SU, Kim BK, et al. Long-term changes of liver stiffness values assessed using transient elastography in patients with chronic hepatitis B receiving entecavir. Liver Int 2014;34:1216–1223.

[9] Fung J, Lai CL, Wong DK, et al. Significant changes in liver stiffness measurements in patients with chronic hepatitis B: 3-year follow-up study. J Viral Hepat 2011;18:e200–e205.

[10] Chon YE, Park JY, Myoung SM, et al. Improvement of liver fibrosis after long-term antiviral therapy assessed by fibroscan in chronic hepatitis B patients with advanced fibrosis. Am J Gastroenterol 2017;112:882–891.

[11] Papatheodoridis GV, Sypsa V, Dalekos GN, et al. Hepatocellular carcinoma prediction beyond year 5 of oral therapy in a large cohort of Caucasian patients with chronic hepatitis B. J Hepatol 2020;72:1088–1096.

[12] Liang LY, Wong VW, Tse YK, et al. Improvement in enhanced liver fibrosis score and liver stiffness measurement reflects lower risk of hepatocellular carcinoma. Aliment Pharmacol Ther 2019;49:1509–1517.

[13] Kim BS, Seo YS, Kim YS, et al. Reduced risk of hepatocellular carcinoma by achieving a subcirrhotic liver stiffness through antiviral agents in hepatitis B virus-related advanced fibrosis or cirrhosis. J Gastroenterol Hepatol 2018;33:503–510.

[14] FDA. Chronic hepatitis B virus infection: developing drugs for treatment. Guidance for industry. Washington DC: US Department of Health and Human Services; 2022.

[15] European Association for Study of Liver; Asociacion Latinoamericana para el Estudio del Higado. EASL-ALEH Clinical Practice Guidelines: non-invasive tests for evaluation of liver disease severity and prognosis. J Hepatol 2015;63:237–264.

[16] de Franchis R, Bosch J, Garcia-Tsao G, et al. Baveno VII - renewing consensus in portal hypertension. J Hepatol 2022;76:959–974.

[17] Arends P, Sonneveld MJ, Zoutendijk R, et al. Entecavir treatment does not eliminate the risk of hepatocellular carcinoma in chronic hepatitis B: limited role for risk scores in Caucasians. Gut 2015;64:1289–1295.
[18] van Kleef LA, Lu Z, Ikram MA, et al. Liver stiffness not fatty liver disease is associated with atrial fibrillation: the Rotterdam study. J Hepatol 2022;77:931–938.
[19] van der Spek DPC, Katwaroe WK, van Kleef LA, et al. Time-trends in disease characteristics and comorbidities in patients with chronic hepatitis B in the period 1980-2020. Eur J Intern Med 2023;107:86–92.
[20] Papatheodoridis G, Dalekos G, Sypsa V, et al. PAGE-B predicts the risk of developing hepatocellular carcinoma in Caucasians with chronic hepatitis B on 5-year antiviral therapy. J Hepatol 2016;64:800–806.
[21] Patmore LA, van Eekhout KMA, Buti M, et al. Hepatocellular carcinoma risk in sub-Saharan African and Afro-Surinamese individuals with chronic hepatitis B living in Europe. J Hepatol 2024;80:243–250.
[22] Sonneveld MJ, Brouwer WP, de Man RA. Hepatocellular carcinoma risk stratification in HBV cirrhosis: time to turn the page? J Hepatol 2020;73:728–729.
[23] Liguori A, Zoncapè M, Casazza G, et al. Staging liver fibrosis and cirrhosis using non-invasive tests in people with chronic hepatitis B to inform WHO 2024 guidelines: a systematic review and meta-analysis. Lancet Gastroenterol Hepatol 2025;10:332–349.

Keywords: Cirrhosis; LSM; Vibration-controlled transient elastography; Advanced chronic liver disease; ACLD.
Received 24 March 2025; received in revised form 5 August 2025; accepted 13 August 2025; Available online 20 August 2025

Journal of Hepatology, Volume *7*

Supplemental information

Persistent risk of hepatocellular carcinoma despite improvement of liver stiffness in patients with chronic HBV with advanced fibrosis

Lesley A. Patmore, Lilian Y. Liang, George Papatheodoridis, Mai Kilany, Arno Furquim d'Almeida, Vincent W.S. Wong, Margarita Papatheodoridi, Thomas Vanwolleghem, Pieter Honkoop, Hans Blokzijl, Özgür M. Koc, Harry L.A. Janssen, Matthijs Kramer, Joep de Bruijne, Apichat Kaewdech, Robert A. de Man, R. Bart Takkenberg, Grace L.H. Wong, Jordan J. Feld, and Milan J. Sonneveld

Persistent risk of hepatocellular carcinoma despite improvement of liver stiffness in patients with chronic HBV with advanced fibrosis

Lesley A. Patmore, Lilian Y Liang, George Papatheodoridis, Mai Kilany, Arno Furquim d'Almeida, Vincent WS Wong, Margarita Papatheodoridi, Thomas Vanwolleghem, Pieter Honkoop, Hans Blokzijl, Özgür M. Koc, Harry L.A. Janssen, Matthijs Kramer, Joep de Bruijne, Apichat Kaewdech, Robert A. de Man, R. Bart Takkenberg, Grace L.H. Wong, Jordan J. Feld, Milan J. Sonneveld

Table of contents

Fig. S1……2

Fig. S2……2

Fig. S3……3

Fig. S4……3

Fig. S5……4

Fig. S1. Flowchart of study inclusion

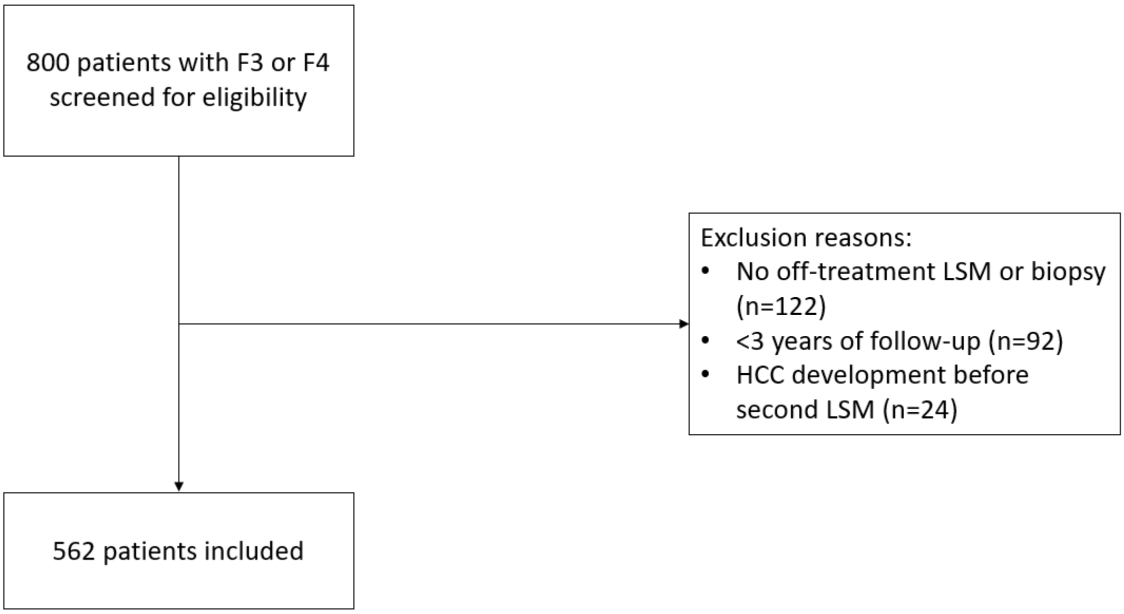

Fig. S2 Cumulative HCC incidence after on-treatment LSM in the overall cohort. *Data was analysed using the Kaplan-Meier method.*

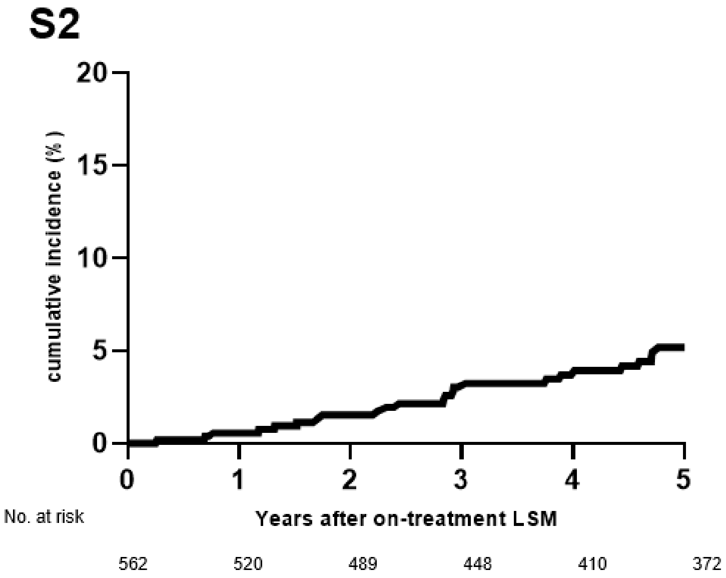

Fig. S3 Cumulative incidence of hepatic decompensation after on-treatment LSM in patients with clinically significant portal hypertension (CPSH) and no CSPH based on the BAVENO VII criteria. *Data was analysed using the Kaplan-Meier method and the log-rank test was used to compare the groups; p ≤0.05 indicaties a statistically significant difference.*

S3

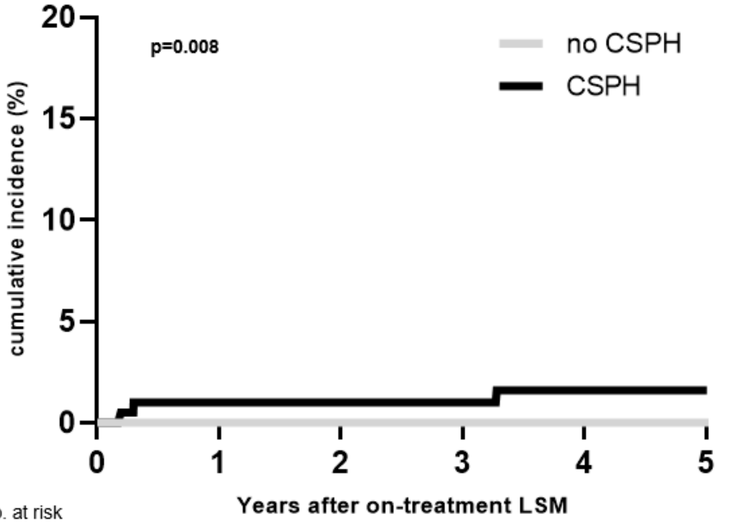

No. at risk						
CSPH	200	188	178	170	158	142
No CSPH	320	294	277	259	235	215

Fig. S4 Cumulative HCC incidence after on-treatment LSM in patients that experienced a >20% decrease in LSM compared to patients with <20% decrease in LSM. *Data was analysed using the Kaplan-Meier method and the log-rank test was used to compare the groups; p ≤0.05 indicaties a statistically significant difference.*

S4

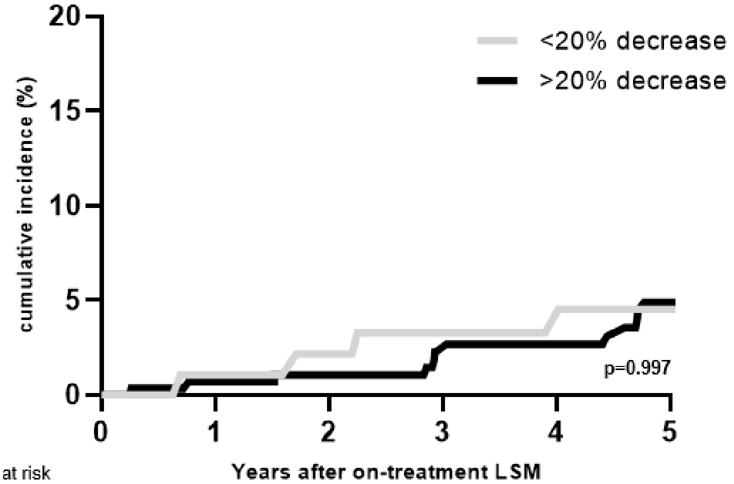

No. at risk						
<20% decrease	102	94	89	82	78	73
>20% decrease	305	277	262	242	223	212

Fig. S5A and 5B Cumulative HCC incidence after on-treatment LSM in patients eligible for HCC surveillance based on (A) PAGE-B score ≥10 and (B) pre-treatment cirrhosis. *Data was analysed using the Kaplan-Meier method and the log-rank test was used to compare the groups; p ≤0.05 indicaties a statistically significant difference.*

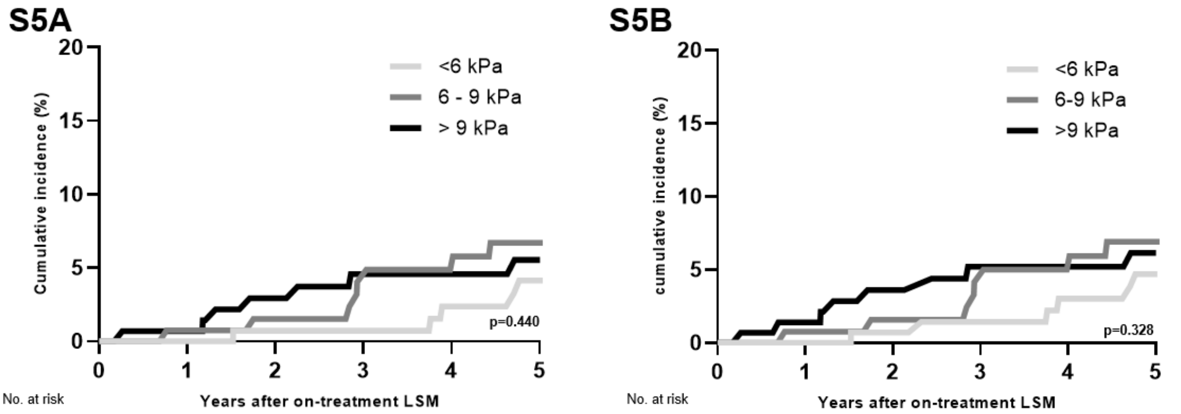

No. at risk						
<6 kPa	153	147	135	130	117	107
6-9 kPa	140	130	128	115	105	97
>9 kPa	147	136	125	113	106	97

No. at risk						
<6 kPa	156	148	138	131	122	112
6-9 kPa	135	125	122	112	102	94
>9 kPa	148	138	127	116	110	98
